# Effects of Percutaneous Electrolysis on Endogenous Pain Modulation: A Randomized Controlled Trial Study Protocol

**DOI:** 10.3390/brainsci11060801

**Published:** 2021-06-17

**Authors:** Sergio Varela-Rodríguez, Juan Luis Sánchez-González, José Luis Sánchez-Sánchez, Miguel Delicado-Miralles, Enrique Velasco, César Fernández-de-las-Peñas, Laura Calderón-Díez

**Affiliations:** 1 Department of Nursery and Physiotherapy, Faculty of Nursery and Physiotherapy, University of Salamanca, 37007 Salamanca, Spain; sergiovarela96@usal.es (S.V.-R.); juanluissanchez@usal.es (J.L.S.-G.); jlsanchez@usal.es (J.L.S.-S.); lauca@usal.es (L.C.-D.); 2 Instituto de Neurociencias de Alicante (CSIC-UMH), 03550 Alicante, Spain; mdelicado@umh.es (M.D.-M.); e.velasco@umh.es (E.V.); 3 Department of Physical Therapy, Occupational Therapy, Physical Medicine and Rehabilitation, Universidad Rey Juan Carlos (URJC), 28922 Madrid, Spain; 4 Cátedra Institucional en Docencia, Clínica e Investigación en Fisioterapia: Terapia Manual, Punción Seca y Ejercicio Terapéutico, Universidad Rey Juan Carlos, 28922 Madrid, Spain

**Keywords:** percutaneous electrolysis, electrical stimulation, ultrasonography, conditioned pain modulation, pain pressure threshold, protocol

## Abstract

Percutaneous electrolysis consists of the application of a galvanic electrical current throughout an acupuncture needle. It has been previously hypothesized that needling procedures’ neurophysiological effects may be related to endogenous pain modulation (EPM). This protocol study describes the design of a double-blind (participant, assessor) randomized controlled trial with the aim to investigate whether percutaneous electrolysis is able to enhance EPM and whether the effect is different between two applications depending on the dosage of the galvanic electrical current. Seventy-two asymptomatic subjects not reporting the presence of pain symptoms the previous 6 months before the study, aged 18–40 years, are randomized into one of four groups: a control group who does not receive any intervention, a needling group who receives a needling intervention without electrical current, a low-intensity percutaneous electrolysis group (0.3 mA × 90 s), and a high-intensity percutaneous electrolysis group (three bouts of 3 mA × 3 s). Needling intervention consists of ultrasound-guided insertion of the needle on the common extensor tendon of the lateral epicondyle. The primary outcome is conditioned pain modulation (CPM), and secondary outcomes include widespread pressure pain sensitivity (pressure pain thresholds (PPT) over the lateral epicondyle, the cervical spine, and the tibialis anterior muscle) and temporal summation (TS). We expected that percutaneous electrolysis would have a greater influence on CPM than an isolated needling procedure and no intervention. In addition, we also postulated that there might be differences in outcome measures depending on the intensity of the electrical current during the percutaneous electrolysis application. This study makes a new contribution to the field of neurophysiological effects of percutaneous electrolysis and needling interventions.

## 1. Introduction

Percutaneous electrolysis is a minimally invasive approach consisting of the application of a galvanic current throughout an acupuncture needle [1,2,3]. The needle is placed directly into soft tissue structures, usually ultra-sound guided. This technique involves the combination of the mechanical stimulation produced by the needle and the electrical/biochemical stimulation provided by the electrical current [4,5,6]. 

Scientific evidence concerning percutaneous electrolysis has increased in the last decade. In fact, a recent meta-analysis has found moderate-quality evidence suggesting a positive effect of percutaneous electrolysis for improving pain and related disability in musculoskeletal pain conditions [7]. One weakness of this meta-analysis was the inclusion of heterogeneous chronic pain conditions, such as patellar tendinopathy, shoulder pain, whiplash syndrome, or temporo-mandibular pain [8,9,10,11,12,13]. 

The exact underlying mechanisms behind the effects of percutaneous electrolysis are not fully understood. It has been proposed that a galvanic current in a saline solution generates a chemical process (“electrolysis”), causing the dissociation of the molecules of sodium chloride and water and producing non-thermal electrochemical ablation [8,14,15]. This organic reaction might be able to stimulate a localized and controlled inflammatory response, hence promoting proper healing of the tissue [5,15,16]. However, this hypothesis is not scientifically supported to date, and although several mechanisms and effects are attributed to percutaneous electrolysis, currently there are few publications on this topic. The application of percutaneous electrolysis in collagenase-induced tendinopathy in rats has been able to produce an increase in expression of anti-inflammatory and angiogenic molecules [16] and an increase in the expression of some genes associated with collagen-remodeling of extracellular matrix [17]. Similar results were found in notexin-induced muscular injury in rats; the application of percutaneous electrolysis produced a decrease in pro-inflammatory mediators and an increase in the expression of anti-inflammatory proteins and vascular endothelial growth factor [15]. In humans, percutaneous electrolysis causes a parasympathetic activity (detected by hearth-rate variability) due to the combination of needle puncture and electric current [1,2]. 

Thus, the exact therapeutic mechanisms of percutaneous electrolysis are not completely defined, and both mechanical and biochemical effects are currently suggested [9]. Recently, it has been hypothesized that dry needling may produce neurophysiological effects integrated into a pain neuroscience paradigm, such as activation of central inhibitory pain pathways, analgesia, conditioned pain modulation, segmental inhibition, or release of endogenous opioids and other neurotransmitters [18,19,20]. These mechanisms should be included in the concept of endogenous pain modulation (EPM), which is the ability of the central nervous system to modulate the nociceptive input from peripheral tissues ascending from the spinal cord to the brainstem [21].

Endogenous pain modulation shows a clinical relevance and plays an important role in the experience of pain. In fact, although the intensity of a nociceptive input is significant for pain, the subsequent modulation of peripheral impulses conditions the intensity of the final painful sensation [22]. In this way, dysfunction of pain inhibitory ability has been associated with chronic pain states such as fibromyalgia, osteoarthritis, or temporomandibular disorders [23]. Further, it has been proposed that EPM efficacy may predict the analgesic effect in response to treatment and that even some therapies are able to potentiate the EPM [24]. No study has investigated the effects of percutaneous electrolysis in EPM. 

The primary aim of this randomized clinical trial is to investigate if the application of percutaneous electrolysis enhances EPM when compared with a simple needle application or no intervention in an asymptomatic healthy population. The secondary aim is to determine if the effects on EPM are different between the application of two different protocols (i.e., low intensity or high intensity of the electrical current) of percutaneous electrolysis. Thus, we first hypothesize that a single session of percutaneous electrolysis will be able to activate EPM in a greater manner than the application of dry needling. Our second hypothesis is that activation of EPM will be higher with the high-intensity protocol than with the low-intensity protocol.

## 2. Materials and Methods

### 2.1. Study Design and Setting

A double-blind randomized controlled trial at the Faculty of Nursing and Physiotherapy, University of Salamanca (Spain) was conducted according to the Consolidated Standards of Reporting Trials (CONSORT) Statement [25]. The current treatment protocol is described according to the recommendations of SPIRIT [26]. 

The protocol of this trial received approval from the Ethics Committee of University of Salamanca (record number 2021/550), and was be conducted according to the Declaration of Helsinki. The clinical trial was registered in ClinicalTrials.gov (registration number NCT04710992).

### 2.2. Participants and Eligibility Criteria 

Participants were recruited from the general population by local advertisements. The participants of this study were asymptomatic adults, aged 18 to 49 years, of both genders, not reporting the presence of pain symptoms the previous 6 months before the study. Volunteers were contacted by email, and those who meet the eligibility criteria were included. All subjects signed a written informed consent before collecting any data.

Participants were excluded if the following were present: 1, belonephobia (fear of needle), 2, neurological, cardiovascular, or metabolic diseases; 3, any medical conditions causing pain; 4, cutaneous alterations; 5, pregnancy; 6, cognitive or sensitivity disorders; 7, fibromyalgia syndrome; 8, frequent/regular consumption of alcohol and other drugs; 9, had received any medication or physical therapy treatment the previous week; 10, intake of caffeine in the two hours prior to measurement; or, 11, vigorous physical activity on the day of testing.

### 2.3. Interventions

This randomized clinical trial had four groups: control group who did not receive any intervention, needling group who received a needling intervention, low-intensity percutaneous electrolysis group who received the intervention for a longer period of time with low intensity of the electrical current (0.3 mA × 90 s), and high-intensity percutaneous electrolysis group who received the intervention for a shorter period of time with high-intensity electrical current (3 bouts of 3 mA × 3 s). All interventions were performed by a physical therapist with more than 10 years of experience in the technique. 

The control group did not receive any intervention for 5 min (time period similar to the remaining groups). The remaining groups received a single session of an ultrasound-guided needle intervention on the common extensor tendon of the lateral epicondyle of their dominant side. Participants were comfortable in a supine position with the dominant elbow on the table in a position of 20° flexion and with the forearm pronated. As described by Rodríguez-Huguet et al. [27], a 25 × 0.3 mm acupuncture needle (Agupunt, Barcelona, Spain) was inserted ultrasound-guided 45° to the skin towards the lateral epicondyle to reach the deep surface of the common extensor tendon. The technique of inserting the needle and the time it is embedded into the participant’s tissues were the same for all groups (90 s) for avoiding different mechanical effect of the needle. The three procedures only differed in the application or not of the electrical current and the intensity of the galvanic current that was applied through the needle. 

The needling group received just the needing intervention without the application of any electrical current. The needle was ultrasound-guided inserted into the common tendon of the lateral epicondyle and left in situ for 90 s without galvanic current. This needling procedure has been previously used in a randomized clinical trial investigating the effects of percutaneous electrolysis in people with plantar heel pain [28].

The low-intensity percutaneous electrolysis group received the intervention for a longer period of time with low intensity of the electrical current. Percutaneous electrolysis was applied using a certified medical device (EPI^®^-Alpha, Barcelona, Spain). Once the needle was ultrasound-guided inserted into the common tendon of the lateral epicondyle, a galvanic current with an intensity of 0.3 mA for 90 s will be applied (total electrical charge applied according to the Coulomb Law: 27 mC). These parameters of electrical current had been previously applied in two previous randomized clinical trials investigating the effect of percutaneous electrolysis in people with shoulder pain [9,10]. 

The high-intensity percutaneous electrolysis group received the intervention for a shorter period of time with high intensity of the electrical current. Percutaneous electrolysis was applied with the same certified medical device as in the low-intensity group (EPI^®^-Alpha, Barcelona, Spain). First, the needle was ultrasound-guided inserted into the common tendon of the lateral epicondyle. During the first 75 s of the application, no electrical current was applied. At the end of the session, three applications of 3 s each at an intensity of 3 mA (total electrical charge applied according to the Coulomb Law: 27 mC) of the galvanic electrical current were applied. These parameters had been previous used in clinical trials investigating the effects of percutaneous electrolysis [12,29]. The round electrode was placed in the lower third of the humerus as closest as possible to the elbow.

### 2.4. Outcomes

All outcomes were evaluated before (2 min) and immediately after (2 min) the intervention by an assessor blinded to the treatment allocation group. The experiment always took place in the same quiet room with a similar temperature and schedule for all participants. Before the baseline outcomes, the following socio-demographic variables were assessed: age (years), gender (male or female), weight (kg), height (m), body mass index (BMI) (kg/m^2^), smoking (yes or no), and level of physical activity (measured by the short version of the International Physical Activity Questionnaire (IPAQ-SF)) [30]. The order of outcome assessment was always the same: widespread PPTs, CPM and TS.

#### 2.4.1. Primary Outcome

The primary outcome was conditioned pain modulation (CPM), which was tested using the upper extremity submaximal effort tourniquet test [31]. Specifically, participants were asked to elevate their non-dominant upper extremity for one minute. A blood-pressure cuff was inflated to a pressure of 280 mm Hg. Participants rated the tourniquet-induced pain intensity of the upper extremity on an 11-point numerical pain rate scale (NPRS, 0–10). The cuff was deflated 6 min after, and 3 trials of pressure pain thresholds (PPTs) at the extensor tendon of the lateral epicondyle, cervical spine, and tibialis anterior muscle were obtained immediately following cuff deflation. Activation of CPM was operationally defined as an increase in PPTs of at least 10% following the conditioning stimulus (tourniquet test). These methods correspond to recommendations on practice of CPM testing [31].

#### 2.4.2. Secondary Outcome

The secondary outcomes included widespread pressure pain sensitivity and temporal summation. All outcomes were assessed by using a digital pressure algometer (Force One FDIX, Wagner Instruments, Greenwich, CT, USA) with a round rubber surface of 1 cm^2^. Participants were supine. Algometry measurements were conducted at the following three locations bilaterally in the following order: common extensor tendon at the lateral epicondyle, tibialis anterior, and cervical spine. These locations were marked with a pen to assess all measurements at the same points.

Widespread pressure pain sensitivity was assessed by determining PPTs bilaterally in the treatment point (elbow), segmental-related point (cervical spine), and a distant non-related point (tibialis anterior). The pressure was applied perpendicular over each point at a rate of 1 kg/s approximately until the subject felt the first sensation of pain [32,33]. At this moment, the algometer was removed and the measurement value was recorded. Participants had two practice trials on the hand before the evaluation begins. Three measurements were obtained at each assessed point with a 30-s interval for avoiding temporal summation [34] and the mean was used for the analysis. If there was a difference of more than 1 kg/cm^2^ between two consecutive measurements at the same point, the highest value was removed and another measurement was obtained.

**Figure 1:**
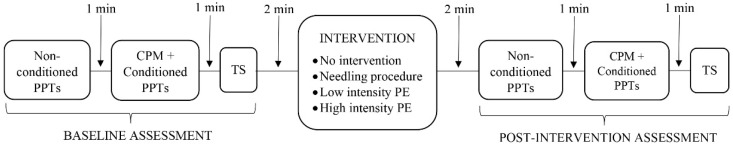
Experimental protocol timeline. PPTs: pressure pain thresholds; CPM: conditioned pain modulation; TS: temporal summation; PE: percutaneous electrolysis.

The evaluation of temporal summation (TS) began one minute after the end of the CPM assessment. The sequential stimulation of mechanical pressure stimuli consisted of 10 pressures with the algometer at PPT level (with 1-s interval between them) on each of the three assessed points, i.e., elbow, neck, and tibialis anterior [35]. The subjects were asked for rating their pain of the 1st and 10th pulse on a numerical pain rating scale (NPRS; 0 = no pain, 10 = maximum tolerable pain). The final rating of TS resulted from the difference between the 10th and the 1st pain rating scores [36]. Figure 1 graphs the experimental protocol timeline.

### 2.5. Sample Size 

The sample size was calculated for the primary outcome. Overall, an increase of at least 10% in PPTs following the conditioning stimulus was considered as an activation of CPM [31]. The determinations were based on detecting pre-post differences of 20% on PPTs following the conditioning stimulus with an alpha level of 0.05, and a desired power of 80%. This generated a sample size of at least 16 participants per group. Similarly, we calculated sample size according to clinically relevant changes. We estimated a large effect size of 1.05 (Cohen’s d) for changes in PPTs following the conditioning stimulus with a statistical power of 80% and an alpha error of 0.05. Therefore, we estimated 16 subjects per group, and considering a 10% lost, 18 individuals per group were recruited (72 subjects in total). GPower 3.1 program was used to calculate the sample size [37].

### 2.6. Allocation and Randomization 

Participants were randomly assigned into one of four intervention groups: control group (no intervention), needling group (receiving a needling intervention but not electrical current), low-intensity percutaneous electrolysis group (receiving percutaneous electrolysis with low intensity of electrical current), and high-intensity percutaneous electrolysis (receiving percutaneous electrolysis with a high intensity of electrical current). 

Concealed allocation was conducted with a computer-generated randomized table of numbers created by an external statistician not involved in the analysis or interpretation of the results. Individual and sequentially numbered index cards with random assignment were prepared. The index cards were placed in sealed opaque envelopes, which were opened by an external assessor. Treatment allocation was revealed to the participants after collection of baseline outcomes. 

### 2.7. Blinding

Both participants and assessors were blinded to the participants group allocation. Due to the nature of the intervention, the clinician could not be blinded. Additionally, the statistical analysis was conducted by an independent statistician who will not aware of the intervention group. 

### 2.8. Statistical Methods 

Data were analyzed by using the IBM-SPSS software package (version 26.0). The significance threshold was established at 0.05 and the limits of the confidence interval at 95%. Changes in outcomes were tested on the basis of the intention-to-treat principle. Firstly, the distribution of normality for all the variables was checked by the Shapiro–Wilk test. Then, a descriptive analysis of baseline characteristics of the sample was reported, calculating the means and standard deviations for those variables with normal distribution and the median and interquartile range for those with non-normal distribution.

Subsequently, to assess the effects of the interventions, we conducted repeated measures analysis of variance (ANOVA) tests for quantitative variables (or Kruskal–Wallis test for non-normal variables) and the Bonferroni test as post hoc comparisons. Mixed-effect regression models considering the group effect, time effect, and group x time interaction were conducted. 

## 3. Discussion

### 3.1. Potential Impact and Significance of the Study

This study hypothesizes that galvanic electrical current applied during percutaneous electrolysis is the responsible of the analgesia-induced produced by this intervention. With that hypothesis, we aim to determine if percutaneous electrolysis has a greater influence in the EPM than an isolated needling procedure or no intervention. Our second hypothesis was that there may be differences in activation of the EPM depending on the intensity and the time of application of percutaneous electrolysis. With that hypothesis, this study will include two different methodological applications (low intensity for a long period of time vs. high intensity for a short period of time) of galvanic electrical current. 

Previous studies have already explored the effects on EPM of different interventions, e.g., shock wave therapy [38], transcutaneous electrical nerve stimulation [39], manual therapy [40,41,42], or exercise [43,44,45]. Thus, this paradigm could potentially explain the analgesic effects of needling interventions, although no study has clarified this either [20]. In fact, EPM has been extensively studied with other treatment techniques resembling percutaneous electrolysis. For example, electroacupuncture also uses an electric current (biphasic wave -but not galvanic- current) through acupuncture needles, and its effects on pain have been related to an activation of descending pain inhibitory systems, but the results are conflicting [46]. In this context, it has been hypothesized that the results depend on the intensity of the electric current, since strong electroacupuncture is able to induce greater analgesic effects than weak electroacupuncture [47]. The effects on pain of other needling techniques, such as acupuncture or dry needling, have also been associated to EPM mechanisms [18,19,20]. Tobbackx et al. [33] conducted a clinical trial similar to the current one proposed here in which one session of acupuncture resulted in acute improvement in PPTs in the neck and calf, but had no effect on CPM or TS in patients with chronic whiplash-associated disorders.

To the best of the authors’ knowledge, there is no published article investigating the effects of percutaneous electrolysis on EPM. In fact, studies that have focused on changes in the nervous system after the application of this intervention found an increased parasympathetic activity, as measured by hearth-rate variability [1,2]. García-Bermejo et al. [1] added a group that received a needling procedure without the galvanic electrical current and found between-groups differences in favor of the application of electrical current throughout the needle. Similar results would be expected in the current study, with both needle and galvanic current influences and differences in favor of the use of an electrical current. Furthermore, we are not aware of any studies comparing the application of percutaneous electrolysis with high and low intensity of the electrical current, so we hope to provide some insight into this particular clinical practice issue.

### 3.2. Limitations

It should be recognized that this protocol has some limitations. First, this study will include healthy participants, which will make it difficult to extrapolate the results to daily clinical practice. Nevertheless, we consider that it is highly important to determine the physiological effects of an intervention such as percutaneous electrolysis first in healthy individuals showing proper CPM and as a second step to determine its effects in different chronic pain conditions, where CPM is usually impaired and, therefore, the response maybe different. Second, another consideration is the inclusion of a non-intervention group to determine the effects of time on CPM. Additionally, although the participants will be blinded from their group assignment, some subjects may perceive discomfort related to the application of galvanic current and identify the intervention that they receive. Successfully blinding will be confirmed by asking the participants which intervention they believed had received. It should be recognized that it will be not possible to blind the therapist who will apply the interventions, considering the treatment approach that is proposed. It would increase the risk of bias due to deviation from intended interventions. Finally, the results of this study will be restricted to the immediate effects of assessments. Since this is a mechanisms-related study, this is the common procedure in published studies [40,41,42].

### 3.3. Contributions to Physical Therapy

The lack of evidence concerning the mechanisms involved in pain reduction after the application of percutaneous electrolysis justifies the development of this randomized clinical trial. In fact, this study will be the first to investigate the influence of percutaneous electrolysis on EPM and its publication will represent a contribution in the field of the neurophysiological effects of needling techniques. In addition, this research may establish new research lines that will include subjects presenting with chronic pain conditions for observing possible differences with the results obtained in healthy subjects. This trial may also contribute to the development of application protocols based on different dosage of percutaneous electrolysis.
